# A Highly Redundant Gene Network Controls Assembly of the Outer Spore Wall in *S. cerevisiae*

**DOI:** 10.1371/journal.pgen.1003700

**Published:** 2013-08-15

**Authors:** Coney Pei-Chen Lin, Carey Kim, Steven O. Smith, Aaron M. Neiman

**Affiliations:** Department of Biochemistry and Cell Biology, Stony Brook University, Stony Brook, New York, United States of America; Duke University Medical Center, United States of America

## Abstract

The spore wall of *Saccharomyces cerevisiae* is a multilaminar extracellular structure that is formed *de novo* in the course of sporulation. The outer layers of the spore wall provide spores with resistance to a wide variety of environmental stresses. The major components of the outer spore wall are the polysaccharide chitosan and a polymer formed from the di-amino acid dityrosine. Though the synthesis and export pathways for dityrosine have been described, genes directly involved in dityrosine polymerization and incorporation into the spore wall have not been identified. A synthetic gene array approach to identify new genes involved in outer spore wall synthesis revealed an interconnected network influencing dityrosine assembly. This network is highly redundant both for genes of different activities that compensate for the loss of each other and for related genes of overlapping activity. Several of the genes in this network have paralogs in the yeast genome and deletion of entire paralog sets is sufficient to severely reduce dityrosine fluorescence. Solid-state NMR analysis of partially purified outer spore walls identifies a novel component in spore walls from wild type that is absent in some of the paralog set mutants. Localization of gene products identified in the screen reveals an unexpected role for lipid droplets in outer spore wall formation.

## Author Summary

The cell wall of fungi is a complex extracellular matrix and an important target for antifungal drugs. Assembly of the wall during spore formation in baker's yeast is a useful model for fungal wall development. The outermost layers of the spore wall are composed of a polymer of dityrosine connected to an underlying polysaccharide layer. The assembly pathway of this dityrosine polymer is not known. Using a genetic approach we reveal a network of genes that function redundantly to control dityrosine layer synthesis. Solid state NMR analysis of spore walls from wild-type and mutant cells reveals a novel constituent of the spore wall that may link the dityrosine to the underlying polysaccharides and a role for lipid droplets in the incorporation of this new component into the spore wall.

## Introduction

All cells surround themselves with some form of extracellular matrix that provides structural integrity to the cell and protection from the environment. While the composition of these extracellular matrices varies, they present all cells with a common problem – how to assemble a complex macromolecular structure in an extracellular milieu. In fungi, the extracellular matrix is referred to as the cell wall and serves as the interface between a fungal cell and its environment. The composition and structure of the wall can determine the ability of cells to survive under different conditions. As a result, cell wall synthesis is an important target for antifungal drugs for the treatment of fungal infections [1].

The cell wall of f`ungi is composed primarily of long chain polysaccharides and heavily glycosylated proteins. In addition, many fungi also contain polyphenolic compounds such as melanin in their walls, though relatively little is known about how these polyphenols are incorporated into the wall [2]. The vegetative cell wall of *Saccharomyces cerevisiae* has been used extensively as a model for studies of fungal cell walls, though it lacks components such as the polyphenols that are found in other fungi [3]. These constituents are found, however, in the wall of *S. cerevisiae* spores.

**Figure 1:**
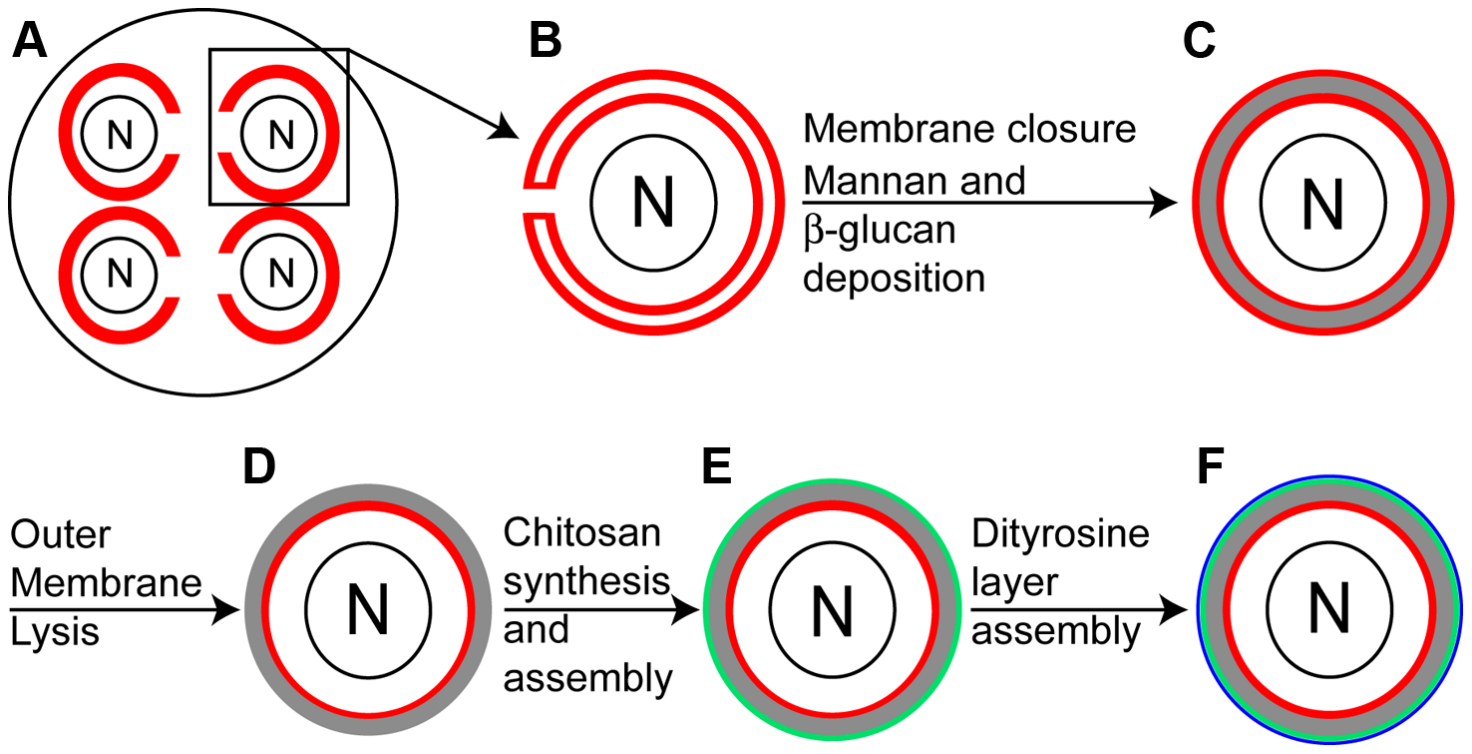
Overview of spore wall formation. A) Each of the four nuclei (N) in a sporulating cell are engulfed by a prospore membrane (red). B) A single prospore membrane prior to closure. C) After closure of the prospore membrane, mannans and β-glucans (gray) are deposited in the lumen between the spore plasma membrane and outer membrane derived from the prospore membrane (both in red). D) The outer membrane disappears, exposing spore wall material to the ascal cytoplasm. E) Chitosan is synthesized and assembled as a discrete layer (green) on the outside of the β-glucan. F) The dityrosine layer (blue) is formed on the outside of the chitosan layer.

Starvation can induce diploid cells of *S. cerevisiae* to undergo meiosis and differentiate to form haploid spores [4]. Spores are formed by an unusual cell division in which four daughter cells are generated within the cytoplasm of the mother cell. Intracellular membranes termed prospore membranes engulf and eventually enclose each of the four nuclei generated by meiosis, giving rise to the daughter cells (Figure 1). Closure of a prospore membrane envelops a nucleus within two membranes; the plasma membrane of the spore and an outer membrane that separates the spore plasma membrane from the mother cell cytoplasm. After prospore membrane closure a spore wall is formed *de novo* around each spore (Figure 1).

The completed spore wall consists of four distinct layers [5]. The first two layers are composed of mannan and β-glucan and are similar in composition to the vegetative cell wall [6]. These two layers are formed in the lumen between the spore plasma membrane and the outer membrane (Figure 1C). After the β-glucan layer is completed, the outer membrane is lost exposing the spore wall directly to the cytoplasm of the surrounding ascus (Figure 1D) [7]. The two outer layers of the spore wall are made from components unique to the spore [8], [9]. First, a layer of chitosan is formed on top of the β-glucan layer after outer membrane lysis (Figure 1E) [7]. Chitosan is a polymer of β-1,4 linked glucosamine moieties and is generated by the combined action of the chitin synthase Chs3 and the sporulation-specific chitin deacetylases Cda1 and Cda2. Chs3 generates and extrudes from the spore chitin, a β-1,4 linked N-acetylglucosamine polymer and the Cda1/2 enzymes then convert the N-acetylglucosamine subunits in the nascent chitin chains to glucosamine [10], [11]. Once assembled, the chitosan layer forms a surface on which the outermost layer of the spore wall assembles. The major component of this outer layer is the cross-linked amino acid N,N-bis-formyldityrosine (hereafter, dityrosine) (Figure 1F) [8].

Dityrosine is formed in the spore cytoplasm by the Dit1 and Dit2 enzymes [12]–[15]. These dityrosine molecules are then moved to the spore wall by the action of transporters localized in the spore plasma membrane. The primary transporter is encoded by *DTR1*; however, *dtr1*Δ cells still display dityrosine fluorescence indicating that alternative transporters exist [16]. Once exported, dityrosine is incorporated into a large, insoluble polymer, the chemical structure of which remains to be determined [9].

Together, the outer spore wall chitosan and dityrosine layers confer enhanced resistance to environmental stresses on the spore, including the ability to pass through the digestive tracts of insects, permitting dispersal in the environment [7], [10], [12]. The composition of the spore wall and its construction in a constrained developmental window make it an excellent model system for the study of fungal cell wall assembly.

Dityrosine in the spore wall is fluorescent and screens for mutants that lack this fluorescence have identified a variety of genes involved in sporulation, including *DIT1* and *DIT2* [12]. However, no genes specifically involved in assembly of the dityrosine polymer have been reported. To search for such genes, a synthetic genetic array approach was used to identify mutants that display reduced dityrosine fluorescence in combination with a mutant in the dityrosine transporter *DTR1*. Double mutant analysis of the genes identified in the primary screen reveal a highly interconnected network of genes contributing to dityrosine assembly. Strikingly, many of the major nodes in this network were found to have paralogs in the yeast genome. For several of these paralogous gene sets, deletion of all the paralogs leads to loss of dityrosine from the spore wall. These results reveal a highly redundant network of genes that regulate the assembly of the dityrosine polymer.

## Results

### A sensitized screen identifies new genes important for dityrosine fluorescence

The transporter Dtr1 functions in the export of dityrosine from the spore cytoplasm, but deletion of *DTR1* results in only a modest reduction in dityrosine incorporation into the spore wall, presumably due to the presence of alternative transporters [16]. We reasoned that extracellular dityrosine might nonetheless be limiting in *dtr1*Δ mutants and so this mutant might be more sensitive to additional perturbations in outer spore wall assembly. Thus, we performed a screen to identify mutants that result in loss of dityrosine fluorescence in combination with *dtr1*Δ.

Using a modified synthetic genetic array protocol a *dtr1*Δ strain was crossed to a collection of knockout mutants [17]. As high sporulation frequency is required, we used a set of 301 knockouts of sporulation-induced genes that was previously constructed in the efficiently sporulating SK1 background [18]. Diploids homozygous for *dtr1*Δ and *mutX*Δ were tested for fluorescence after sporulation on plates by a patch assay. Fluorescence was monitored by sporulating cells on nitrocellulose filters and then exposing the filters to shortwave UV light. For each gene, homozygous *mutX*Δ *DTR1* diploids were also examined using the same assay. Mutants that displayed wild-type fluorescence in the *DTR1* background but reduced fluorescence in combination with *dtr1*Δ in at least two of three replicates, were considered to have a genetic interaction with *dtr1*Δ.

Mutants in 38 genes that displayed reduced dityrosine fluorescence only in combination with *dtr1*Δ were identified (Table 1). Most of these genes were not identified in previous screens of this same collection of knockouts in SK1 for other phenotypes associated with outer spore wall defects including ether sensitivity, spore wall permeability, and sensitivity to digestion by *Drosophila* indicating that the synthetic screen worked to uncover new genes with possible roles in outer spore wall assembly [7], [19], [20]. Several of the mutants identified were in uncharacterized ORFs with no gene designation. For reasons explained below, we have named some of these ORFs. The acronym for *OSW6* (*IRC18*/*YJL038w*) and *OSW7* (*YFR039c*) stands for Outer Spore Wall), while the acronym for *LDS1* (*YAL018w*), and *LDS2* (*YOL047c*) stands for Lipid Droplet in Sporulation.

**Table 1:** Mutants displaying synthetic dityrosine defects with *dtr1*Δ.

ORF	Gene	Found in earlier spore wall screens^a^	Comments^b^
*YAL018c*	*LDS1*		paralog of *LDS2*
*YBR134w*		Ether	dubious ORF/overlaps *HSL7*
*YBR250w*	*SPO23*		
*YCR010c*	*ADY2*		acetate transporter
*YDL076c*	*RXT3*		HDAC complex subunit
*YDR108w*	*TRS85*		TRAPP subunit
*YDR273w*	*DON1*		leading edge complex protein
*YDR480w*	*DIG2*		
*YDR506c*	*GMC1*		synaptonemal complex assembly
*YDR525w*	*API2*		dubious ORF
*YEL016c*	*NPP2*		nucleotide pyrophosphorylase
*YER106w*	*MAM1*	Permeable	kinetochore protein
*YER182w*	*FMP10*		mitochondrial protein
*YFR023w*	*PES4*		RNA binding motif
*YFR039c*	*OSW7*	Fly	signal peptide
*YGL096w*	*TOS8*		putative transcription factor
*YGL144c*	*ROG1*		putative lipase
*YHR153c*	*SPO16*		synaptonemal complex assembly
*YIR013c*	*GAT4*		putative transcription factor *YJL037w*
*YJL037w*	*IRC18*/*OSW6*	Fly	paralog of *OSW4*
*YJL038c*	*LOH1*/*OSW4*	Permeable	paralog of *OSW6*
*YJL162c*	*JJJ2*		DNAJ domain
*YJR036c*	*HUL4*		E3 ligase
*YJR099w*	*YUH1*		ubiquitin hydrolase
*YKL121w*	*DGR2*		
*YLL030c*	*RRT7*		dubious ORF
*YLL054c*			
*YLR030w*			
*YLR238w*	*FAR10*		FAR complex subunit
*YLR373c*	*VID22*	Ether	
*YML119w*			
*YMR147w*			
*YMR148w*	*OSW5*	Permeable	
*YMR262w*			
*YOL047c*	*LDS2*		paralog of *LDS1*
*YOR350c*	*MNE1*		mitochondrial protein
*YPL033c*	*SRL4*	Permeable	
*YPL184c*	*MRN1*		RNA binding

a Whether a mutation in the gene was identified in earlier screens of the same collection for ether sensitive spores [7], spores with permeable spore walls [20], or spores with lowered resistance to digestion by *Drosophila* [19].

b Information from the SGD: www.yeastgenome.org.

### A highly redundant network of genes is involved in dityrosine layer assembly

A significant fraction of the mutants examined in the screen (13%) displayed fluorescence defects in the sensitized *dtr1*Δ background. This suggests that the process is somehow buffered so that individual mutants have only modest effects. One possible explanation for the failure of individual mutants to produce strong effects on dityrosine would be the presence of redundant pathways leading to dityrosine incorporation. If so, combining mutants in genes involved in two separate pathways might lead to decreased dityrosine fluorescence even in the presence of *DTR1*. This hypothesis was tested by constructing double mutants between genes identified in our screen and examination of dityrosine fluorescence.

As gene products directly involved in assembly of polymerized dityrosine are likely localized to the spore wall, we focused on those with predicted signal peptides or transmembrane domains, or with predicted enzymatic activities. In addition, we also included the genes *OSW3* and *YEL023c* since these genes have been implicated in outer spore wall formation in other studies [our unpublished results and [20]]. Most double mutant combinations were constructed twice and each strain was tested in triplicate for dityrosine fluorescence. Double mutants exhibiting a reduction in dityrosine fluorescence in at least two of the three replicates were scored as a genetic interaction (Table S1).

**Figure 2:**
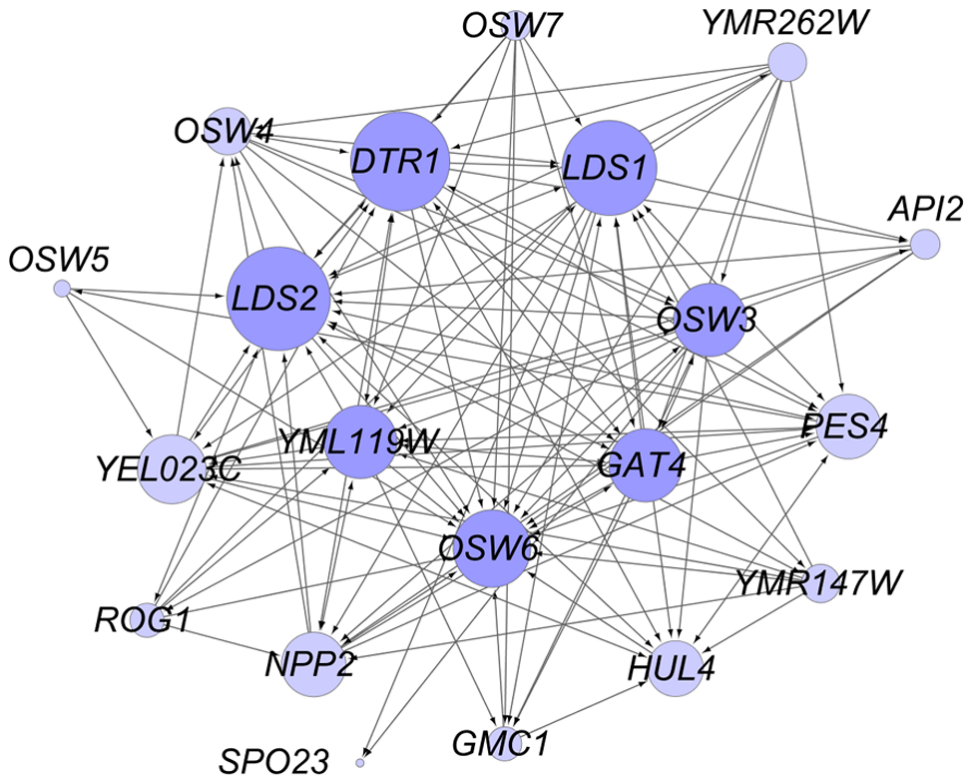
Genetic interactions between genes involved in dityrosine layer formation. Cytoscape was used to represent the genetic interactions listed in Table S1. Lines between the circles indicate reduction of dityrosine fluorescence in the double mutant. Double mutants were constructed from two independent pairs of parental strains and tested in three biological replicates (see Methods). Arrowheads indicate whether the interaction was seen in one (one arrowhead) or both (two arrowheads) double mutant strains. Circles in darker purple indicate genes with >10 interacting partners. The size of the circles increases with the number of genetic interactions seen for each gene.

Reduced dityrosine fluorescence was observed for many different mutant combinations. Most of the mutants tested displayed interactions with at least seven other mutants. The extent of these interactions can be seen in graphic representation of the interaction network which shows a highly interconnected set of genes with relatively few outliers having limited synthetic interactions (Figure 2). This architecture suggests that assembly of the dityrosine layer is accomplished through a number of alternative processes with overlapping function. Major hubs of the network, such as *OSW6* or *LDS2*, show interactions with most of the other genes and might represent particularly important functions in assembly of the outer spore wall.

### Paralogous genes are redundant for dityrosine assembly

**Figure 3:**
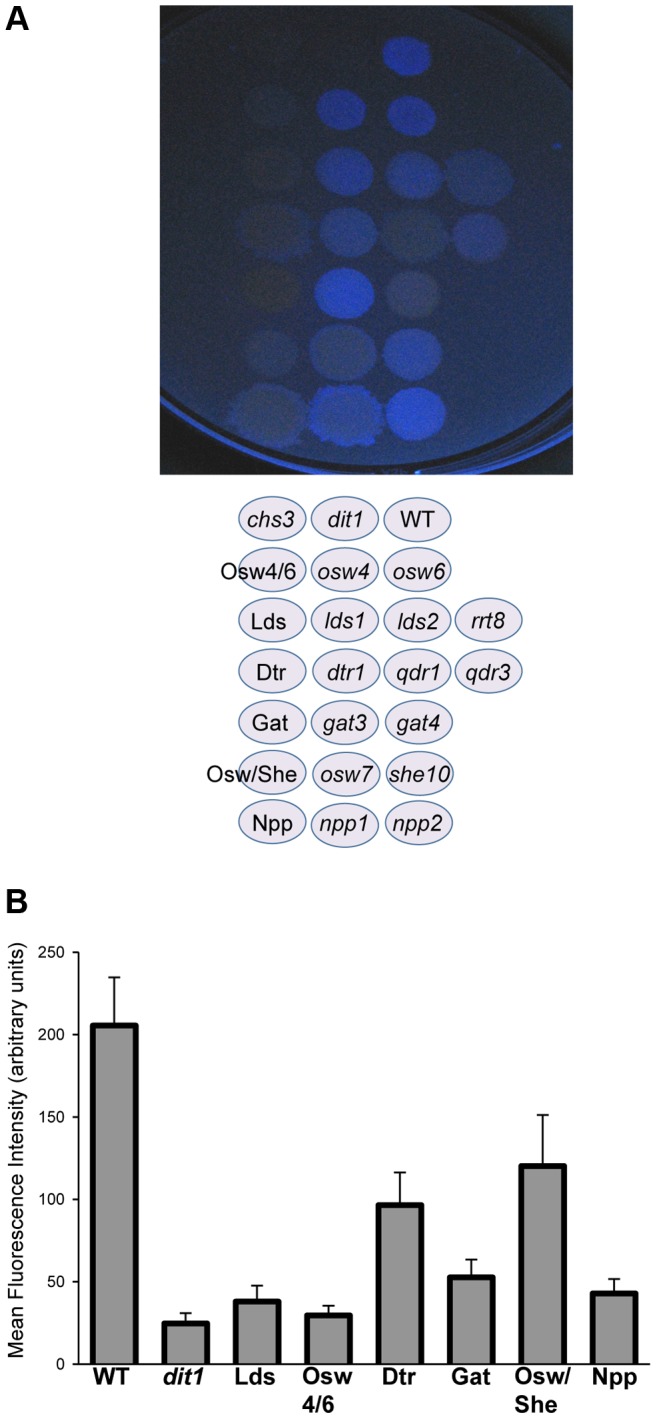
The effect of deletion of paralog sets on dityrosine fluorescence. A) Patch assay of dityrosine fluorescence. Single and multiple mutant diploid cells of the indicated genotypes were sporulated on filters and the filter was exposed to UV light to detect dityrosine fluorescence. B) Quantitation of dityrosine fluorescence in the paralog mutant strains. Dityrosine fluorescence from the spore wall was quantified for strains of the indicated genotypes using a fluorescence microscopy assay. For each strain, the fluorescence value represents the average of twenty individual cells. Error bars indicate one standard deviation. Multiple mutant diploids are indicated by the name of the set of genes deleted as listed in Table 2.

The double mutant analysis suggests that different processes can compensate for each other in spore wall assembly. Another type of redundancy results when different genes encode proteins with the same function. Of the 20 genes shown in the network in Figure 2, 11 are genes with potential paralogs in the *S. cerevisiae* genome (Table 2). If the paralogous genes are functionally redundant, then mutation of all the genes within a set should lead to a more severe phenotype. This idea was tested by construction of diploids homozygous for deletions of all of the genes within the first six paralog sets listed in Table 2 (attempts to construct deletions for the other three sets were unsuccessful). These diploids were then examined for dityrosine fluorescence both by patch assay and quantitatively using fluorescence microscopy [20] (Figure 3). Multiple mutants in all six of the paralog sets displayed reduced dityrosine fluorescence relative to single mutants, supporting the idea that these genes encode functionally redundant proteins. Quantitatively, dityrosine fluorescence was reduced by at least 45%, in deletions of all the paralog sets. The most severe defects were seen in the *lds1*Δ *lds2*Δ *rrt8*Δ and *osw4*Δ *osw6*Δ mutant strains where the fluorescence signal was reduced to the levels of a *dit1*Δ, which lacks dityrosine.

**Table 2:** Paralogous gene sets implicated in outer spore wall formation.

ORF	Gene	Sporulation-induced^a^	% Similarity^b^
**Lds set:**			
YAL018c	*LDS1*	Y	53% w/*RRT8*
YOL047c	*LDS2*	Y	38% w/*LDS1*
YOL048c	*RRT8*	N	58% w/*LDS2*
**Osw4/6 set:**			
YJL038c	*OSW4*/*LOH1*	Y	50% w/*OSW6*
YJL037w	*OSW6*/*IRC18*	Y	
**Dtr set:**			
YBR180w	*DTR1*	Y	59% w/*QDR3*
YIL120w	*QDR1*	N	48% w/*DTR1*
YBR043c	*QDR3*	N	48% w/*QDR1*
**Gat set:**			
YLR013w	*GAT3*	Y	70% w/*GAT4*
YIR013c	*GAT4*	Y	
**Osw/She set:**			
YFR039c	*OSW7*	Y	48% w/*SHE10*
YGL228w	*SHE10*	N	
**Npp set:**			
YCR026c	*NPP1*	N	57% w/*NPP2*
YEL016c	*NPP2*	Y	
**Fet set:**			
YDR506c	*GMC1*	Y	52% w/*FET5*
YMR058w	*FET3*	N	50% w/*GMC1*
YFL041w	*FET5*	Y	65% w/*FET3*
**Rog set:**			
YGL144c	*ROG1*	Y	63% w/*ROG1*
YDL109c		N	
**Pes set:**			
YFR023w	*PES4*	Y	59% w/*MIP6*
YHR015w	*MIP6*	Y	

a Indicates whether the transcript is induced during sporulation [55].

b % similarity is based on comparison of the two sequences using BLASTP [56].

### Paralogous genes are not required for the presence of a chitosan layer

The deletion of the paralogous pairs (and triples) is distinct from the combination with *dtr1*Δ or other mutants in that these mutations likely remove a single function (e.g., Osw4/6 function) rather than weakening two different aspects of assembly (e.g., Osw4 function and Lds1 function). For this reason, the strains carrying deletions of all the genes for a particular paralog set were phenotypically characterized to determine what role each paralogous group plays in outer spore wall assembly. For simplicity, each multiple mutant strain will be indicated by the name of the paralog set given in Table 2, e.g. the *lds1*Δ *lds2*Δ *rrt8*Δ strain will be referred to as the Lds mutant.

**Figure 4:**
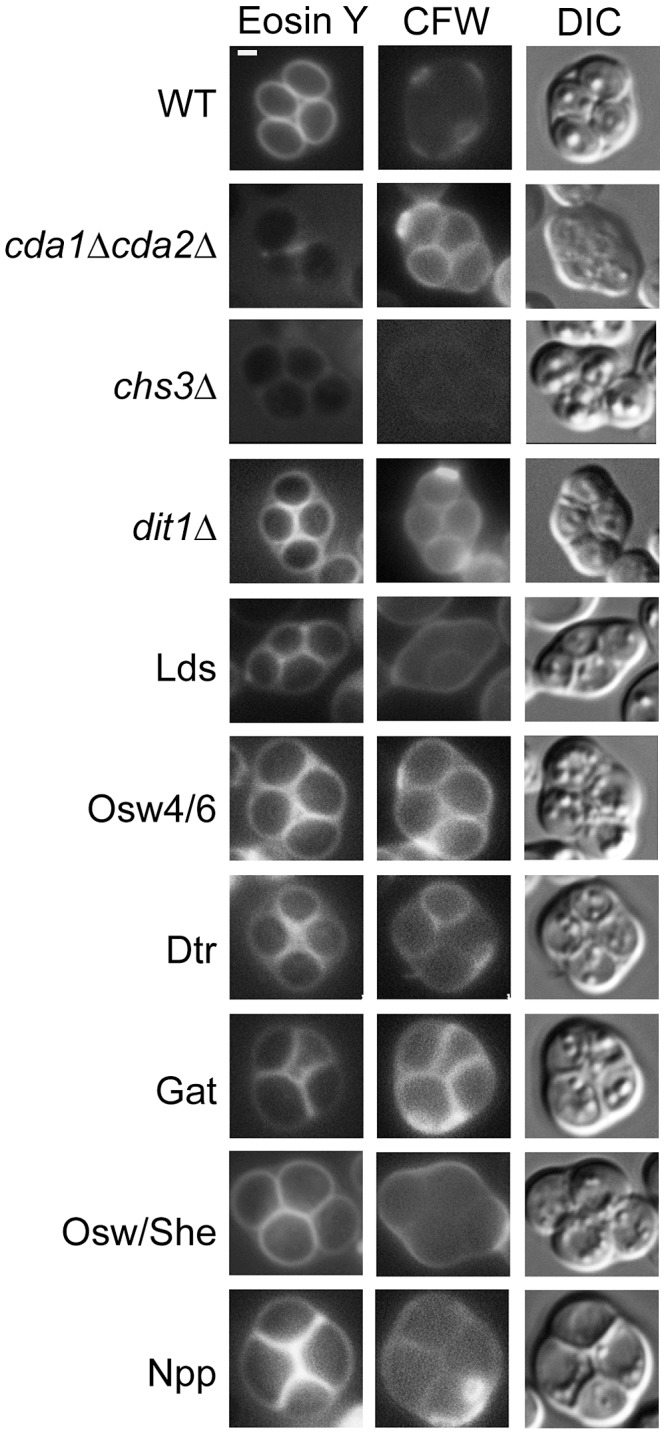
The effect of deletion of paralog sets on chitosan. Strains of the indicated genotype were sporulated and stained with both Eosin Y and Calcofluor White (CFW) to visualize the chitosan layer. The *cda1*Δ *cda2*Δ, *chs3*Δ, and *dit1*Δ strains provide standards for the conditions chitin/no chitosan/no dityrosine, no chitin/no chitosan/no dityrosine, and chitin/chitosan/no dityrosine, respectively. For all strains >30 cells were examined and >60% display the staining patterns shown here. Quantitation for each strain is given in Table S2. DIC = differential interference contrast. Scale bar = 1 micron. Multiple mutant diploids are indicated by the name of the set of genes deleted as listed in Table 2.

Assembly of the dityrosine layer requires the underlying chitosan layer, so loss of dityrosine fluorescence could be an indirect effect of loss of chitosan [10]. Each paralog set mutant strain was stained with the chitin/chitosan binding dye Calcofluor White as well as the chitosan specific dye Eosin Y and examined in the fluorescence microscope. In wild-type cells, spores stain weakly with Calcofluor White because the dityrosine layer blocks the dye from binding the underlying chitosan [21]. By contrast, Eosin Y binds efficiently to the chitosan in the wild-type spores [22] (Figure 4, top row). Deletion of the *CDA1* and *CDA2* genes encoding chitin deacetylases results in spore walls that contain chitin instead of chitosan, resulting in the absence of a dityrosine layer [11]. Consistent with this fact, *cda1*Δ *cda2*Δ spores stain with Calcofluor White but not with Eosin Y (Figure 4, 2^nd^ row). A *chs3*Δ mutant lacks both chitin and chitosan and as a result stains with neither dye (Figure 4, 3^rd^ row). A *dit1*Δ mutant contains chitosan but lacks dityrosine and binding of both dyes is seen (Figure 4, 4^th^ row).

None of the paralog sets is required for formation of the chitosan layer, as all of the paralogous mutant strains stained with Eosin Y. However, staining of the Lds mutant strain was noticeably less intense than in *dit1*Δ, suggesting that this mutant may have some loss of chitosan in addition to dityrosine. Compared to wild type, the Lds, Osw4/6, Dtr, and Gat mutants all displayed increased Calcofluor White staining consistent with reduced levels of dityrosine in the wall. The Osw/She and Npp strains did not show clear increases in Calcofluor White staining. For Osw/She this is perhaps consistent with the quantitatively modest reduction in dityrosine fluorescence in the double mutant (Figure 3B).

### Paralogous genes are required for functional spore walls

**Figure 5:**
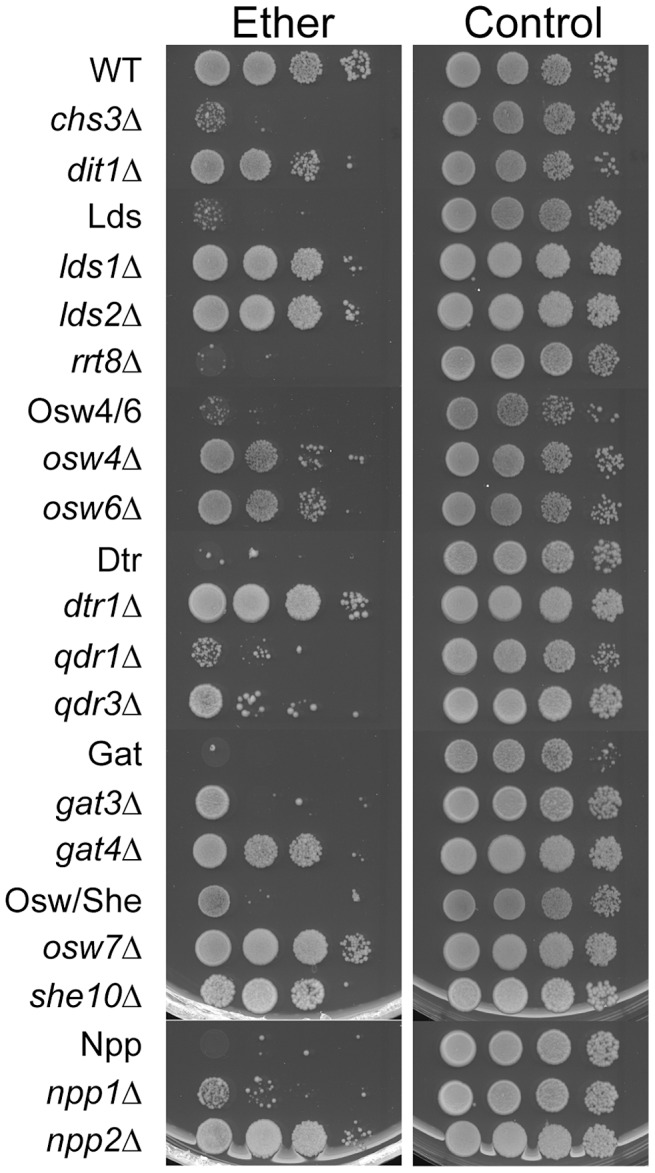
The effect of deletion of paralog sets on ether resistance of spores. Strains of the indicated genotypes were sporulated in liquid to >70% asci and then 10-fold serial dilutions were spotted onto YPD plates. Left panels, plates exposed to ether vapor for 45 minutes before incubation at 30°C. Right panels, no ether control. Multiple mutant diploids are indicated by the name of the set of genes deleted as listed in Table 2.

Spore walls function to protect cells form various exogenous stresses. One assay that is commonly used for spore wall function is sensitivity to ether vapor. Ether is toxic to vegetative cells but the outer layers of the spore wall confer resistance to ether exposure [23]. To test whether the various paralog sets are required not just for wild-type levels of dityrosine fluorescence but also for making functional spore walls, diploids containing deletions of each set were tested for ether sensitivity. The outer layers of the spore wall contribute to ether resistance, but to different extents so that *dit1*Δ mutants, which lack dityrosine, are sensitive but less so than *chs3*Δ mutants, which lack both chitosan and dityrosine (Figure 5). All of the paralog set mutants showed increased sensitivity to ether. In fact, all of the combinations appeared more sensitive to ether than a *dit1*Δ strain, suggesting that the spore wall defect in these mutants may be more significant than just the loss of dityrosine. In the case of *the* Osw4/6 and Osw/She mutants, deletion of both paralogs is necessary to reveal the sensitive phenotype. In the other paralog sets, however, deletion of one of the genes is sufficient to account for much of the sensitivity. This is most obvious in the Lds set where the *rrt8*Δ single mutant is as ether sensitive as the *lds1*Δ *lds2*Δ *rrt8*Δ triple mutant. The patterns of dityrosine fluorescence in the individual mutants (Figure 3A) do not necessarily reflect the patterns of ether sensitivity. Thus, the mechanistic link between ether resistance and outer spore wall structure is unclear. However, the ether tests reveal that the spore walls in all of the paralog mutants are compromised in their ability to confer stress resistance to the spore.

### Solid state NMR identifies a novel component of the spore wall

**Figure 6:**
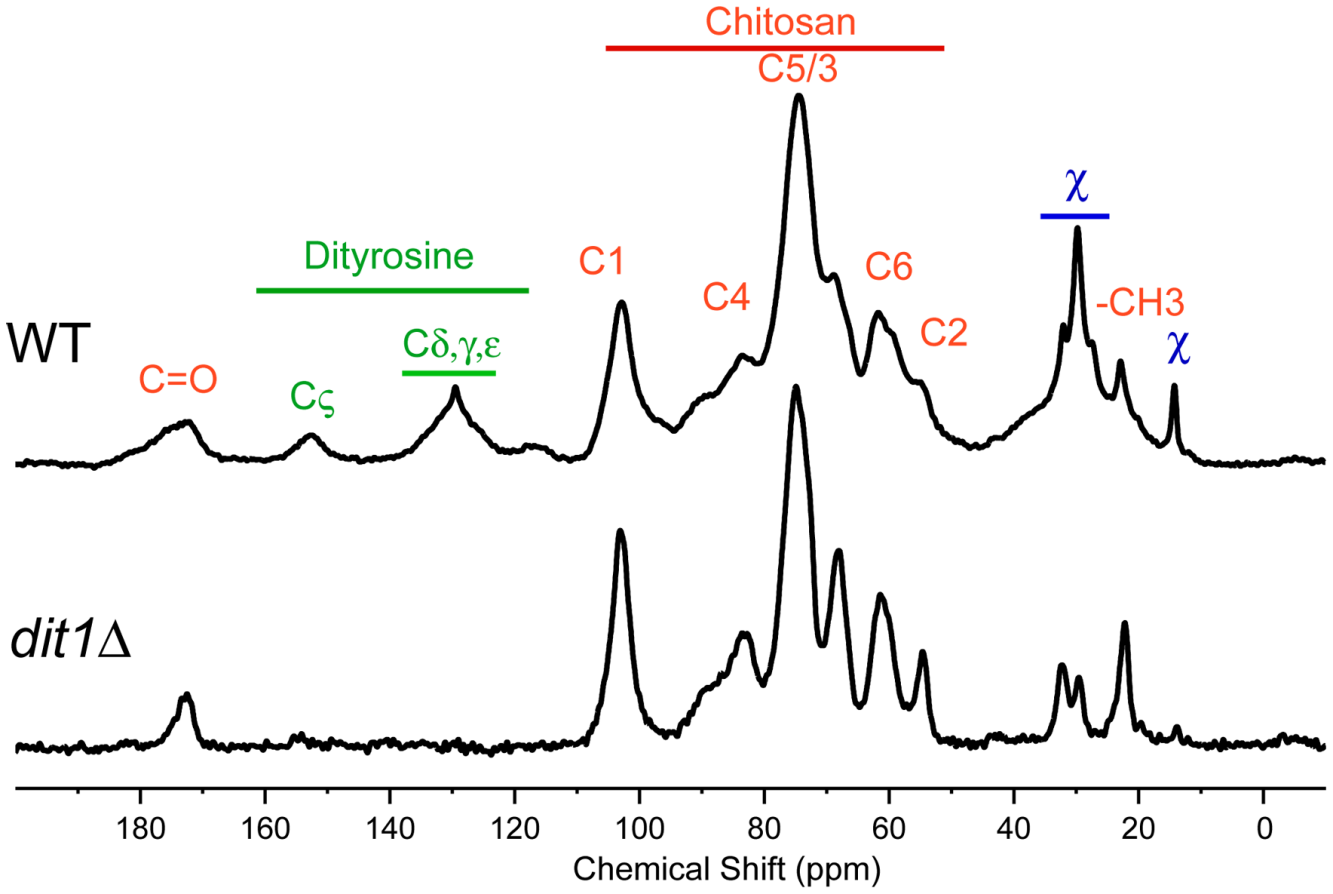
NMR analysis of the outer spore wall. Outer spore walls were purified from wild type (AN120) or *dit1*Δ cells and then analyzed by solid state ^13^C NMR. Resonances assigned to chitosan carbons are designated by red labels and resonances from dityrosine are indicated by green labels. Unassigned chemical shifts from component χ are indicated in blue. Upper spectrum, spore wall from wild type. Lower spectrum, spore wall from *dit1*Δ. For comparison, spectra are scaled so the C5/3 resonance of chitosan is of constant height.

The hypothesis that multiple assembly routes are used for dityrosine incorporation into the spore wall suggests that different mutants might accumulate different intermediates in the biosynthetic pathway. Solid-state nuclear magnetic resonance (NMR) has been proven to be an effective tool for the analysis of intact bacterial cell walls [24], [25] and we sought to apply this technology to analyze the spore wall. An established protocol for purification of spore walls was adapted to isolate large quantities of spore wall fragments enriched for just the outer spore wall layers. These purified outer spore walls from wild type spores were analyzed using ^13^C solid state NMR spectroscopy. The samples were not isotopically enriched but relied on the natural abundance of ^13^C in the wall components. The ^13^C spectrum from a wild-type spore wall prepared in this way is shown in Figure 6. The identities of many of the chemical shifts were confirmed by comparison to spectra prepared using purified chitosan and tyrosine (Figure S1). The strongest resonances are from the six carbons of the glucosamine ring of chitosan (labeled in red in Figure 6). The conversion of chitin to chitosan is incomplete during spore wall formation as shown by the unique resonance at 22 ppm that corresponds to the methyl carbon of the acetyl moiety on N-acetyl-glucosamine groups present in the polymer (indicated by –CH3 in Figure 6). The carbonyl resonance associated with the N-acetyl-glucosamine groups is also observed at ∼174 ppm, but overlaps with the carbonyl resonance of dityrosine. A similar incomplete conversion of chitin to chitosan has been reported in cell walls of other fungi [26]. Small resonances at chemical shifts between 130 ppm and 155 ppm (labeled in green) correspond to the carbons of the dityrosine rings as confirmed by the loss of these signals when the spore wall of a *dit1*Δ mutant was examined (Figure 6, lower spectrum). As expected, the resonances corresponding to chitosan (and the residual N-acetyl-glucosamine groups of chitin) are still observed in the *dit1*Δ spore walls. In the spectrum of the *dit1*Δ mutant, the integrated intensities of carbonyl and methyl resonances of N-acetyl-glucosamine groups is close to 1∶1, indicating that the shoulder on the C = O resonance in the wild-type spectrum is primarily due to dityrosine. In addition, a number of significant resonances in the range of 25 to 35 ppm were observed that are not from chitosan or dityrosine and, thus, represent an unknown component of the outer spore wall, which we designate as component χ (labeled in blue) (Figure 6).

### Solid state NMR reveals distinct outer spore wall defects in the paralog mutants

**Figure 7:**
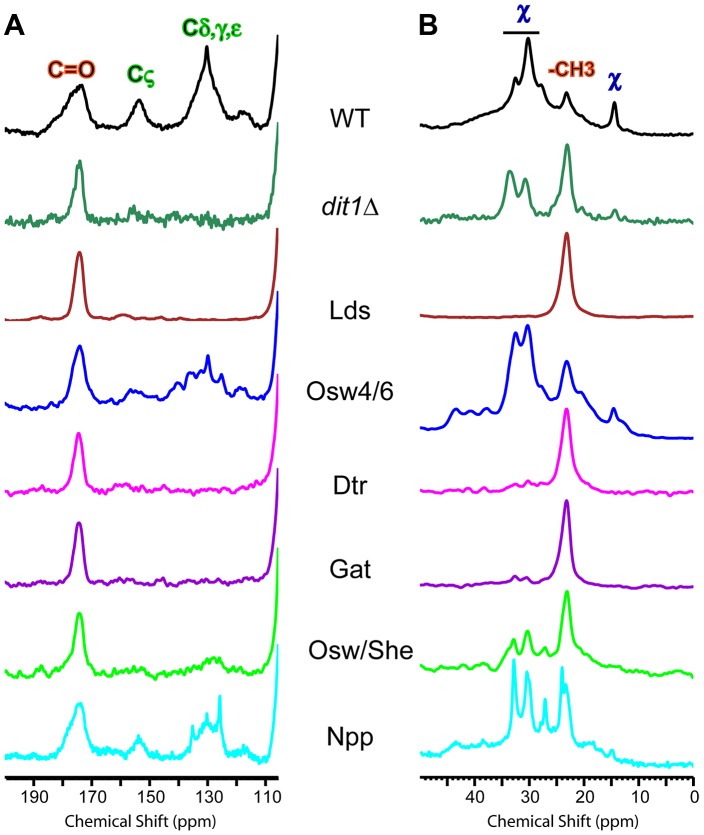
NMR analysis of outer spore walls in the paralog mutant strains. A) The dityrosine region of the ^13^C NMR spectrum is shown for spore walls from wild type, *dit1*Δ and the indicated paralog mutant strains. Positions of the dityrosine chemical shifts and chitin carbonyl chemical shifts are indicated above the wild type spectrum. The spectra have been scaled so the height of the carbonyl resonance is constant. B) The component χ region of the ^13^C NMR spectrum is shown for spore walls from wild type, *dit1*Δ and the indicated paralog mutant strains. Positions of the component χ chemical shifts and chitin methyl group shifts are indicated above the wild type spectrum. The spectra have been scaled to the C5/3 resonance as in Figure 6. Multiple mutant diploids are indicated by the name of the set of genes deleted as listed in Table 2.

Spore walls prepared from six different paralog mutant strains were similarly analyzed by solid state NMR. Complete spectra for each strain are shown in Figure S2. The dityrosine and component χ regions of the spectra are shown in Figure 7. The relative heights of the carbonyl and C_ζ_ resonances can be used to assess the amount of dityrosine in the spore wall (Figure 7A). For component χ, the height of the resonances relative to the -CH_3_ peak provides an indicator of its abundance (Figure 7B). The phenotypes of the various mutants fell on a spectrum with respect to their severity: the Npp mutant showed no strong effect on dityrosine or component χ incorporation; the Osw4/6 mutant had reduced dityrosine but not component χ incorporation; the Osw/She mutant showed reduced dityrosine and a modest reduction component χ incorporation; the Gat and Dtr mutant strains had strong reductions in both dityrosine and component χ; and the Lds mutant appeared to completely lack resonances for both dityrosine and component χ This NMR analysis confirms the effect of the paralog set mutants on dityrosine incorporation. In addition it identifies a new component of the outer spore wall, and shows that the Lds family proteins are essential for incorporation of both dityrosine and component χ into the wall.

### The Lds proteins are localized to a specific population of lipid droplets

**Figure 8:**
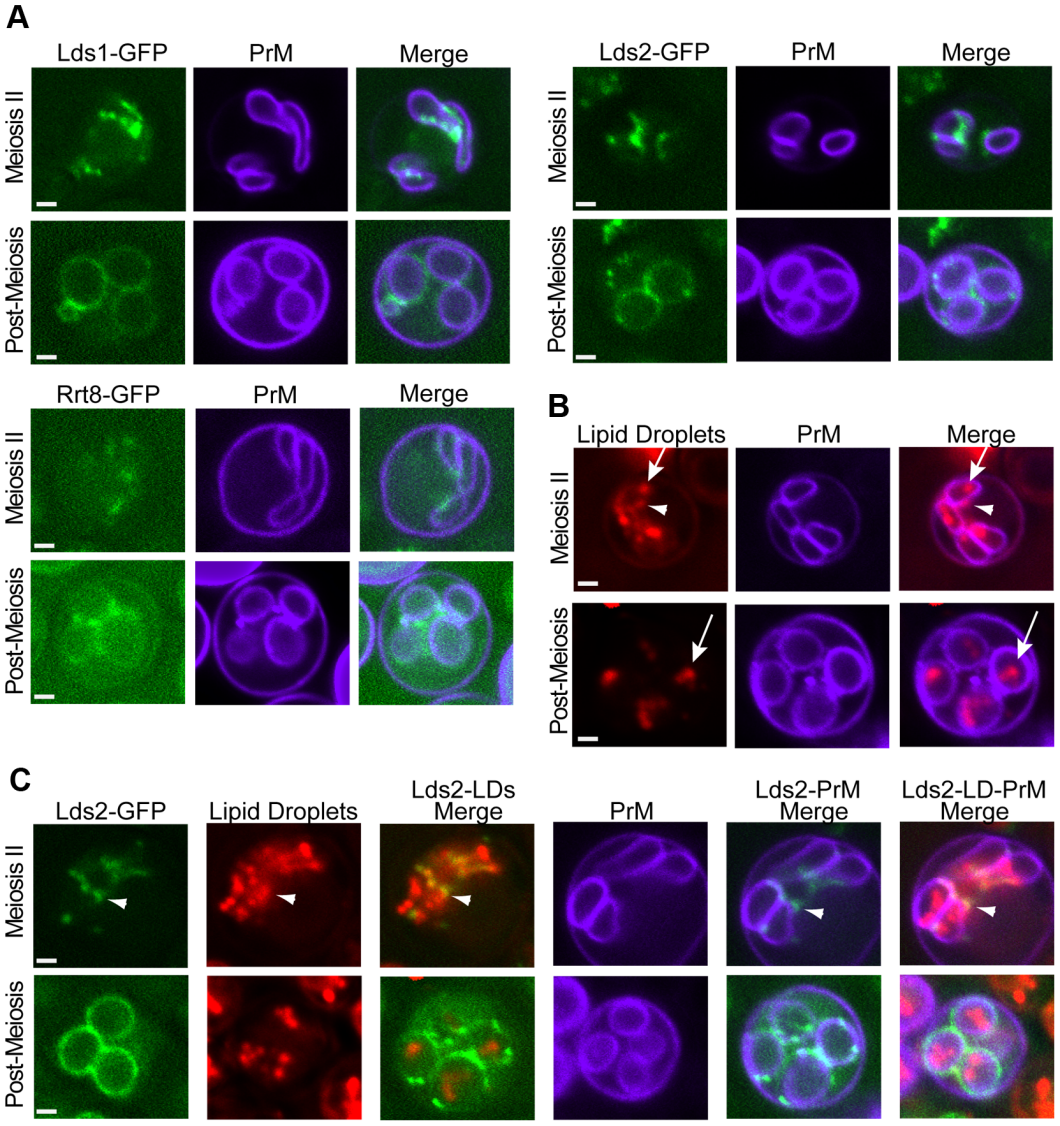
Localization of the Lds proteins involved in outer spore wall synthesis. A) Diploids expressing GFP fusions to the indicated protein as well as mTag-BFP-Spo20^51–91^ as a prospore membrane (PrM) marker were sporulated and examined by fluorescence microscopy. Examples of a cell in the middle of Meiosis II and a post-meiotic cell are shown. B) Sporulating wild-type cells expressing mTag-BFP-Spo20^51–91^ were stained with the lipid droplet marker Bodipy TR. Arrowheads indicate lipid droplets associated with the ascal side of the prospore membrane. Arrows indicate lipid droplets within the prospore membrane C) Cells expressing both Lds2-GFP and mTag-BFP-Spo20^51–91^ were stained with Bodipy TR. Arrowheads indicate localization of Lds2-GFP to a lipid droplet outside of the prospore membrane. For all experiments at least 20 cells at the indicated stage were scored and 100% of the cells displayed patterns similar to those shown here. Quantitation for each experiment is given in Table S2. Scale bars = 1 micron.

The possible role of the Lds proteins in component χ synthesis led us to examine the localization of these proteins. In sporulating cells, Lds1-GFP, Lds2-GFP, and Rrt8-GFP displayed similar localizations, concentrating in discrete patches or puncta along the ascal sides of the growing prospore membranes in cells in mid-Meiosis II. In post-meiotic cells, the proteins localized more uniformly around the outside of spores, consistent with localization to the spore wall (Figure 8A). To assess the functionality of the fusion protein, an *RRT8*-*GFP* strain was mated to an *rrt8*Δ, the resulting diploid was sporulated, and the ether sensitivity of the spores was examined. *RRT8*-*GFP* partially complemented the ether sensitivity of the *rrt8*Δ mutant, indicating that the fusion protein is at least partially functional and suggesting that the localization is relevant to Lds function (C. Lin, unpublished observations).

An Rrt8-GFP fusion protein has previously been reported to localize to lipid droplets in vegetative cells, and an association of lipid droplets with the prospore membrane has been previously reported in electron microscopy studies [27]. To determine if the foci observed for Lds set proteins during sporulation are lipid droplets, the localization of this organelle in sporulating cells was investigated. BODIPY 493/503, which has a green fluorescence, can be used to stain lipid droplets in yeast [28]. However, because the Lds proteins are fused to GFP that also fluoresces green, an alternative method for visualizing lipid droplets was required for co-localization experiments. Red fluorescent BODIPY TR displayed an identical staining pattern to BODIPY 493/503 in vegetative yeast cells making it a good alternative to BODIPY 493/503 (Figure S3). When cells in Meiosis II were stained with BODIPY TR, bright staining lipid droplets were seen inside of the prospore membrane with smaller but clear staining of droplets outside of the prospore membrane as well (Figure 8B). In post-meiotic cells, lipid droplets are seen inside of the spores, but the BODIPY TR staining outside of the prospore membrane is lost. As BODIPY stains lipid droplets by partitioning into the hydrophobic core of the droplet, this loss of staining suggests that the lipid constituents of the droplets outside of the prospore membrane are consumed during the process of spore wall development.

To determine whether the Lds proteins are present in lipid droplets, cells expressing both a blue fluorescent marker for the prospore membrane and Lds2-GFP were sporulated and stained with BODIPY TR. In mid-Meiosis II cells, Lds2-GFP puncta overlapped with or were directly adjacent to a subset of the lipid droplets that appear to be outside of the prospore membrane. No localization of Lds2-GFP to lipid droplets inside of the prospore membrane was observed (Figure 8C). In post-meiotic cells, no overlap of GFP with BODIPY TR staining was seen, indicating that the lipid droplets to which Lds2-GFP localizes are the ones consumed during spore wall formation. Thus, the Lds proteins localize to a specific subset of developmentally regulated lipid droplets.

**Figure 9:**
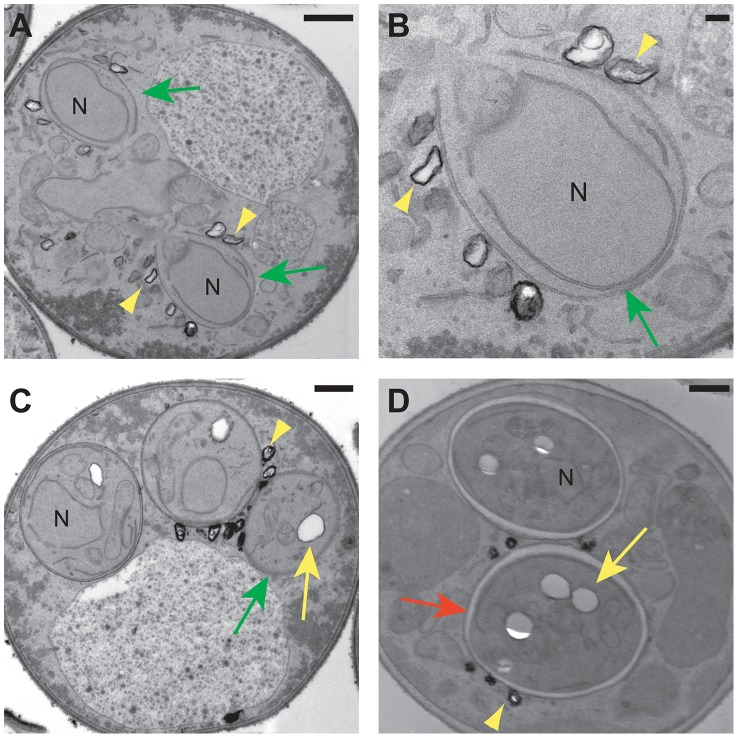
Electron microscopy of lipid droplets in sporulating cells. Electron micrographs of wild type cells. A) A cell in Meiosis II showing two prospore membranes (green arrows) engulfing nuclear lobes (N). Lipid droplets can be seen associated with the prospore membrane (yellow arrowheads). Scale bar = 500 nm. B) Higher magnification of one of the prospore membranes in A. Scale bar = 100 nm. C) Post meiotic cell showing dark-stained lipid droplets (yellow arrowhead) associated with the outer membrane (green arrow). Lipid droplets inside of the spore (yellow arrow) do not stain as darkly with permanganate. N = nucleus. Scale bar = 500 nm. D) Lipid droplets in the ascus (yellow arrowhead) associated with the outer spore wall (red arrow) of mature spores. Yellow arrow indicated a lipid droplet within the spore cytoplasm. N = nucleus. Scale bar = 500 nm.

The fluorescence images suggest that the specific lipid droplets to which the Lds proteins localize are found on or outside of the prospore membrane. However, the resolution of the fluorescence images is not sufficient to clearly determine the position of the lipid droplets relative to the prospore membrane. Transmission electron microscopy was used to examine the behavior of lipid droplets in sporulating wild type cells at higher resolution (Figure 9). Consistent with the fluorescence experiments, in mid-Meiosis II cells, lipid droplets can bee seen in close contact with the side of the prospore membrane facing the presumptive ascal cytoplasm (Figure 9A, B). These droplets are often irregularly shaped and their surface stains more darkly with permanganate than droplets inside of the prospore membrane. After prospore membrane closure, lipid droplets remain associated with the outer membrane (Figure 9C). In more mature asci, where spore wall assembly is nearly complete, lipid droplets in the ascal cytoplasm are less numerous, but can still be found associated with the outer spore wall after outer membrane lysis (Figure 9D). Thus, there is a sub-population of lipid droplets that remain associated with the exterior surface of the spore throughout spore wall formation. The localization of the Lds proteins to these lipid droplets positions the proteins so that they may contribute directly to outer spore wall morphogenesis.

## Discussion

Many screens for sporulation defective mutants in *S. cerevisiae* have been published and yet very few genes have been reported to affect the incorporation of dityrosine into the spore wall, which has been a significant obstacle to studies of wall development [18], [29]–[32]. This work shows that two levels of genetic redundancy are utilized in dityrosine assembly, which provides an explanation for the failure of earlier mutant screens to reveal the genes involved. The first level of redundancy is a redundancy of process in which mutations in two genes not related by sequence is required to reveal a phenotype. In these instances, the gene products, e.g., the secreted protease Osw3 and the ubiquitin ligase Hul4, have unrelated functions and yet the presence of one somehow buffers the loss of the other. How this is achieved is unclear. One possibility is that dityrosine might be incorporated into the wall via multiple mechanisms, for example different chemical linkages, and that loss of any individual linkage is insufficient to significantly reduce the fluorescence of the wall.

The second level is redundancy of function; many of the genes involved have paralogs of overlapping activities present in the genome and so deletion of multiple paralogs is required to produce a strong phenotype. Paralogous genes are relatively common in *S. cerevisiae* due to a whole genome duplication event that occurred during the evolution of *Saccharomyces* [33]. Of the paralogs listed in Table 2, five pairs (*LDS1*/*LDS2*, *ROG1*/*YDL109c*, *NPP1*/*NPP2*, *OSW7*/*SHE10*, *PES4*/*MIP6*) can be assigned to the whole genome duplication [34].

Paralogs with overlapping function are predicted to be lost from the genome without selective pressures for retention of both copies [35]. Several explanations have been advanced to account for redundant paralogs, including compensatory regulation, dosage effects, and incomplete specialization [35]. In particular, redundant genes involved in development in metazoans have been suggested to serve as a buffering system to protect against error [36], [37]. A similar logic may account for the redundancy in spore wall development. Many of the genes involved in spore wall development function only during this phase of the yeast life cycle and, therefore, cannot be selected for during mitotic growth. Redundancy of these activities may provide “insurance” against loss-of-function mutations arising during vegetative growth. By this reasoning, one might expect increased redundancy amongst genes involved in sporulation. It is notable in this regard that targeted deletion of sporulation-induced genes in *S. cerevisiae* revealed obvious phenotypes for only 11% of the mutants [18]. Similar studies in *S. pombe* reveal a somewhat higher percentage (30%), but again the majority of sporulation-induced genes display no deletion phenotype, suggesting that redundancy might be a common feature of developmental processes in yeast [38], [39]. Synthetic mutant analysis is well suited to address the genetics of redundant systems. The application of SGA analysis, as presented here, should be a valuable tool for dissecting other aspects of sporulation.

### Functions of the paralog sets

What specific roles do these different gene products play in assembly of the outer spore wall? For several of the genes identified, the function of the proteins is likely indirect. For example, Dtr1 and related transporters are required for delivery of precursors for assembly. Similarly, the Gat3 and Gat4 proteins are DNA-binding proteins and, thus, are likely to be required for transcription of other genes whose products act on assembly. Very similar consensus binding sites have been defined for both Gat3 and Gat4 [40]. A search of yeast promoter sequences with these consensus sequences failed to identify any of the genes implicated in outer spore wall assembly (A. M. N., unpublished observations). Identification of the target genes for this pair of transcription factors may, therefore, identify additional genes involved in spore wall development.

Of the genes identified by the screen, *OSW4*/*OSW6* and *OSW7*/*SHE10* are the best candidates to encode proteins directly involved in assembly of the dityrosine layer. These genes encode proteins with predicted signal peptides and GFP fusions to Osw4 localized to the prospore membrane consistent with spore wall localization (C. Lin and A. M. Neiman, unpublished observations). Moreover, the ^13^C NMR spectra of both of the Osw4/6 and Osw/She mutants are similar to that of *dit1*Δ, showing reduced dityrosine, but significant levels of both chitosan and component χ. Thus, these mutants appear somewhat specific in their effects on the dityrosine. It remains to be determined if these proteins play an enzymatic or structural role in the wall.

### A role for lipid droplets in spore wall assembly

In vegetative cells, lipid droplets are seen as multiple puncta near the nuclear envelope and ER [28]. This distribution is changed in sporulating cells where a subset of lipid droplets is associated specifically with the ascal side of the prospore membrane. The Lds proteins are found on this class of lipid droplets and are essential for outer spore wall assembly, revealing a function for lipid droplets in this process.

These results also suggest the existence of distinct sub-populations of lipid droplets, as defined both by localization and protein composition, during sporulation. Analysis of different lipid droplet proteins in mammalian cells has also revealed discrete sub-populations of lipid droplets in some cell types [41], [42]. Recruitment of distinct classes of droplets for specific functions may be a conserved behavior for this organelle.

The role of the Lds proteins in spore wall assembly is unclear, though the localization of lipid droplets to the surface of the spore wall (after outer membrane lysis) means that the Lds proteins could play a direct role in assembly. In addition to lacking dityrosine, the Lds mutant completely lacks component χ and has reduced Eosin Y and Calcofluor White staining, suggesting that the levels of chitosan may be reduced as well. Thus, the effect on dityrosine may be secondary to these other deficiencies, for instance, incorporation of component χ may be necessary for subsequent dityrosine assembly. Perhaps, some of the reactions in spore wall assembly actually occur on the surface of the lipid droplet, aided by the Lds proteins, before final incorporation of components into the wall. Additionally, both the EM studies and BODIPY staining suggest that the lipid constituents of the prospore membrane-associated lipid droplets are consumed during the course of spore wall assembly. Whether these lipids are used as an energy source for the spore, to expand the prospore membrane, or in some other way contribute to spore wall morphogenesis remains to be determined.

### NMR analysis reveals a new component of the spore wall

Our results demonstrate that solid state NMR is an effective assay to examine the composition of the outer spore wall. The ^13^C spectrum of the outer spore wall is not overly complex, containing ∼20 distinct carbon resonances. This relative simplicity suggests that more sensitive 2-dimensional NMR assays could be effective in defining the structural organization of the spore wall, including how the different components are linked to each other.

In addition, our analysis reveals a previously unknown component of the outer spore wall, which we designate χ. Based on peak height of the NMR resonances, component χ is less abundant in the wall than chitosan, but more abundant than dityrosine. From the analysis of the *dit1*Δ spore wall it is clear that incorporation of component χ into the wall does not require dityrosine. Moreover, the Lds mutant spectrum shows that the chitosan layer is still formed in the absence of component χ. These data raise the possibility that component χ might act as a linker between the chitosan and dityrosine components of the spore wall.

The chemical nature of component χ remains to be determined, though based on previous biochemical characterizations of purified outer spore walls, it seems unlikely to be either a polysaccharide or a protein [9]. The lack of a carbonyl resonance associated with component χ in the NMR data also indicates that it does not contain amino acids. The positions of the component χ chemical shifts are consistent with reduced carbons such as in alkanes. Given that the Lds proteins required for component χ incorporation are localized to lipid droplets adjacent to the developing spore wall, perhaps generation of component χ requires material that is delivered from the lipid droplets to the spore wall.

It is also noteworthy that levels in the spore wall of component χ are reduced in the *dtr1*Δ *qdr1*Δ *qdr3*Δ mutant strain. This strain lacks dityrosine in the wall because of a failure to export monomeric dityrosine from the spore cytoplasm [16]. However, the reduction of component χ in this strain cannot be an effect of reduced dityrosine because incorporation of component χ is unaffected in *dit1*Δ cells that lack dityrosine. One straightforward possibility is that like dityrosine, some precursor to component χ is synthesized in the spore cytoplasm and exported to the wall by Dtr1 and related transporters.

### Implications for other fungi

Genomic sequencing has revealed that many pathogenic fungi possess the enzymes for the production of chitosan and dityrosine. This is true even of fungi that do not form spores, such as *Candida albicans*. In fact, dityrosine has been found in the vegetative cell walls of *C. albicans* and mutants in dityrosine synthesis show drug sensitivity consistent with cell wall defects [43], [44]. Chitosan is also an important component of the wall of the pathogen *Cryptococcus neoformans*, where it is required for cell wall integrity and for virulence [22], [45]. While *Cryptococcus* does not synthesize dityrosine, it incorporates an analogous polyphenolic compound, melanin, into the cell wall and melanization is important for resistance of the fungus to various environmental stresses and may play an important role in the evasion of host immune responses during infection [2], [46]. Interestingly, *C. neoformans* mutants lacking chitosan display a “leaky melanin” phenotype, suggesting that chitosan is required for proper incorporation of melanin into the cell wall [22]. The nature of the connection between the carbohydrate and polyphenol components of the spore wall is, therefore, an important general issue in understanding the structure of the fungal cell wall. Assembly of the outer spore wall in *S. cerevisiae* provides a tractable model to address this issue. Our results identify several new genes intimately involved in construction of the outer spore wall. Further analysis of these gene products should provide insights into the structure of the spore wall and the mechanisms of its assembly.

## Methods

### Strains and media

Yeast strains used in this study are listed in Table S3. Unless otherwise indicated, standard yeast media and growth conditions were used [47]. For synthetic medium containing Geneticin (G418), monosodium glutamate (Sigma) was added as a nitrogen source instead of ammonium sulfate. Drug concentrations used were 200 mg/L G418, 3 mg/L cycloheximide, 1 g/L 5-FOA, and 300 mg/L Hygromycin B. Strain CL62 was derived from the *ybr180w*Δ strain from the systematic yeast knockout collection [48] by selection for growth on plates containing cycloheximide. To construct the paralog deletion strains (CL6, CL7, CL15, CL26, and CL57) *lds1*Δ, *dtr1*Δ, *gat4*Δ, *osw7*Δ, and *npp2*Δ strains from the SK1 knockout collection [18] were crossed to *rrt8*Δ, *qdr3*Δ, *gat3*Δ, *she10*Δ, and *npp1*Δ strains from the BY4741 knockout collection [48], respectively. After sporulation and dissection of the resulting diploids, double mutant haploids were identified by marker segregation, confirmed by PCR, and CL15, CL26 and CL57 were constructed by mating of appropriate segregants. The single gene deletion strains CL38, CL43, CL44, CL47, and CL59 were also obtained as segregants from these crosses. All knockout alleles were confirmed by PCR. For construction of CL6 and CL7, deletion of the third paralog was achieved by PCR-mediated gene disruption in double mutant haploids [49]. For CL6, primers LDS2-KO-F/LDS2-KO-R (for primer sequences see Table S4) and pAG32 [50] as template were used to generate deletion of *LDS2*. For CL7, primers QDR1-KO-F and QDR1-KO-R were used to delete *QDR1*. Single deletions of *OSW4* and *OSW6* (strains CL52 and CL54) were generated using the primer sets ANO262-A/ANO262-B for *OSW4* and ANO263-A/ANO263-B for *OSW6* and pFA6a-HIS3MX6 as the template to generate deletions in strains AN117-4B and AN117-16D followed by mating of the haploids. Simultaneous deletion of both *OSW4* and *OSW6* (strain CL35) was achieved using the same strategy but ANO262 and ANO263 were used as primers. CL50 was constructed by using the primers CDA1&2-KO-F and CDA1&2-KO-R to simultaneously delete both *CDA1* and *CDA2* in AN117-4B and AN117-16D.

### Plasmids

Prospore membranes were visualized using pRS426-Spo20^51–91^-mTagBFP, which expresses a fusion of amino acids 51 to 91 of Spo20 to the N-terminus of mTagBFP under the control of the *TEF2* promoter. To construct this plasmid, a yeast codon-optimized version of mTagBFP [51] flanked by *Pac*I and *Asc*I sites was synthesized (GeneWiz Inc., New Jersey) and cloned into pUC57. A *Pac*I-*Asc*I fragment was then used to replace the mCherry sequence in pRS426-Spo20^51–91^-mCherry.

### Screen for synthetic interactions with *dtr1*Δ

A 48-pin replicator was used to transfer a collection of 301 deletions of sporulation-induced genes in the SK1 background [18] onto 90 mm YPD plates. All strains were arrayed in triplicate. Because the strains in this collection are *HO*, the arrayed patches were replica plated to SPO plates to generate haploid spores. CL62 cells (*MAT* ***a*** *dtr1*Δ *cyh2*) were crossed to these haploid spores by replica plating the sporulated patches to YPD plates spread with CL62. Diploids from this cross were selected on SD-Ura -Trp -His plates. To obtain double deletion mutants, the patches were then transferred to SPO medium and then to SD(MSG) -His +G418 +cycloheximide +5-FOA plates. On this medium, -His and G418 select for the presence of the two knockouts while the cycloheximide and 5-FOA select against any unsporulated diploid cells. Patches containing the double deletion cells were allowed to grow and spontaneously diploidize (all patches should contain both mating types due to the presence of the *HO* gene) and then replica plated onto nitrocellulose membranes on SPO plates for dityrosine fluorescence detection under UV_302_.

### Construction of double mutants for pairwise interactions

To examine synthetic interactions between different genes implicated in outer spore wall assembly, individual strains carrying deletions of each gene examined (listed in Table S1) were taken from the SK1 knockout collection and crossed to a set of strains from the BY4741 knockout collection carrying deletions of all the remaining genes analyzed. Each double mutant combination was constructed twice, once with each gene as the *MAT* **a** parent in the initial mating (i.e. from the yeast knockout collection) and once with each gene as the *MATα* parent (from the SK1 knockouts). Construction of the initial diploids, isolation of the double mutants, and scoring of dityrosine fluorescence was performed similarly to the *dtr1*Δ synthetic screen described above except that no selection for cycloheximide resistance was used. Three separate isolates for each SK1 knockout were crossed to the other deletions and reduction of dityrosine fluorescence intensity in two of the three replicates was considered a synthetic interaction.

### Visualization for pairwise genetic interaction networks

The network in Figure 2 was created using Cytoscape (version 2.8.3) [52].

### Analysis of dityrosine fluorescence by patch assay

Cells to be assayed were grown as patches on YPD plates for 1 day and then replica-plated onto nitrocellulose membranes (Whatman Optitran BA-S 85) on YPD plates. After two days growth, the membranes were transferred to SPO plates and incubated for 3 days at 30°C. The membranes were then transferred to petri dishes containing 200 µl of water, 50 µl of 10 mg/ml zymolyase and 15 µl of β-mercaptoethanol at 30°C for 5 hrs. Finally, the membranes were wetted with 0.1 M NaOH solution to raise the pH and exposed to short wave UV light for the detection of dityrosine fluorescence.

### Microscopy and image processing

All images were collected on a Zeiss Axio-Observer Z1 microscope using a Hamamatsu ER-G camera. Images were collected and fluorescence intensity was measured using Zeiss Axiovision software (version 4.7).

#### Quantitative analysis of dityrosine fluorescence

Dityrosine fluorescence from the spore wall was quantified as described [20]. Briefly, cells were sporulated in liquid SPO medium for three days, centrifuged, washed once with water, re-suspended in 5% ammonia solution to raise the pH, and finally transferred onto microscope slides. Images were collected with a dityrosine fluorescence-specific filter set (Ex. 320 nm/Em. 410 nm) at fixed exposure times. The dityrosine fluorescence intensity (in arbitrary units) was calculated for each cell by taking the average intensity at two points in the spore wall and subtracting the background fluorescence outside of the spore.

#### Calcofluor White and Eosin Y staining

Sporulated cells were harvested and washed with 1 ml McIlvaine's buffer (0.2 M Na_2_HPO_4_/0.1 M citric acid [pH 6.0]) followed by staining with 30 µl Eosin Y disodium salt (Sigma) (5 mg/ml) in 500 µl McIlvaine's buffer for 10 min at room temperature in the dark. Cells were then washed twice in McIlvaine's buffer to remove residual dye and resuspended in 200 µl McIlvaine's buffer. One microliter of a 1 mg/ml Calcofluor White solution (Sigma) was then added to the Eosin Y-stained cells before transfer to microscope slides. Fluorescence of Calcofluor White and Eosin Y stains was examined using DAPI and FITC filter sets, respectively.

#### BODIPY TR methyl ester staining

Sporulating cells were stained with 5 µM of BODIPY TR methyl ester (Life Technologies) in PBS buffer at room temperature for 10 min and washed twice with PBS buffer. BODIPY TR fluorescence was visualized using a Rhodamine filter set.

### Electron microscopy

Sporulating yeast cells were collected and processed for electron microscopy as described in [53].

### Test of ether resistance

Cells were sporulated in liquid SPO medium at 30°C for 2 days. All cultures displayed at least 75% sporulation. Serial dilutions (1, 10^−1^, 10^−2^, 10^−3^) of sporulated cells were spotted on two YPD plates. One plate served as an ether-negative control. The other plate was inverted over ether-soaked filter paper (Whatman #3, 1003-090) for 45 min. Plates were incubated at 30°C for one to two days and photographed.

### NMR methods

#### Spore wall sample preparation

Isolation of spore walls was performed similarly to that previously described [54]. ∼25 g (wet weight) of sporulated wild type (AN120) cells were collected (2.5 L of SPO medium at 75% sporulation frequency), resuspended and washed with 0.1 M sodium phosphate buffer (pH 7.2) and then resuspended in 60 ml of 0.1 M sodium phosphate buffer (pH 7.2) containing 20 mg of Zymolyase (US Biologicals, Salem, MA) as well as 0.04% of ß-mercaptoethanol. The suspension was incubated with shaking at 30°C for 2 hr until spores were released from asci. To remove ascal debris, spore pellets were washed three times at 4,000 rpm for 10 min in a swinging bucket rotor with 0.5% Triton X-100 solution and then resuspended in a small volume (around 10 ml) of 0.5% TX-100, layered onto a 30 ml Percoll (MP Biomedicals, Santa Ana, CA) step gradient [Percoll/TX-100/2.5 M sucrose; from bottom to top 0.8/0.0005/0.1→0.7/0.001/0.1→0.6/0.0015/0.1→0.5/0.002/0.1] and spun at 10,000 rpm for 45 min at 4**°**C in a Sorvall SS34 rotor to separate spores from vegetative cells. Spores were collected from the bottom layer and washed three times with 0.5% TX-100 at 6,000 rpm for 5 min. The collected spores were then mixed with 20 ml of 0.5% Triton X-100 solution and 20 ml of 0.5 mm zirconium/silica beads (BioSpec, Bartlesville, OK) for lysis by bead beating. Spores were disrupted in a BeadBeater (BioSpec, Bartlesville, OK) with pulses of 20 sec ON/40 sec OFF until 80% of spores were disrupted (as determined by light microscopy). The spore wall fragments were separated from intact spores and spore cytoplasm by layering onto a cushion of 60% Percoll/2% TX-100/H_2_O and centrifugation at 23,000 rpm in a Beckman SW41 rotor at 4**°**C for 1 hr. Spore wall fragments form a visible band within the Percoll cushion and this band was collected, the Percoll diluted by addition of 35 ml of 0.5% Triton TX-100 and the fragments were pelleted by centrifugation at 6,000 rpm in a Sorvall SS-34 rotor for 5 min. The wall pellets were then washed three times with distilled H_2_O. Spore walls from mutant strains appear to have a somewhat lower density and so the concentration of Percoll used in the purifications had to be adjusted. For AN264, CL35, CL7, CL15, and CL26, the steps in the Percoll gradient at the first stage were 0.5→0.4→0.3→0.2. The cushion used in the second stage was 40% Percoll/2% TX-100/H_2_O. For CL6, the Percoll gradient was 0.35→0.25→0.15→0.05 and the Percoll cushion was 35% Percoll/2% TX-100/H_2_O.

To remove the inner spore wall layers, collected spore wall fragments were first resuspended in 5 ml of 50 mM Tris (pH 8.0) containing 1% SDS and 5 mM DTT. The sample was heated at 95°C for 1 hr and then 1 mg of protease K (US Biologicals) was added and the solution incubated at 50°C for 24 hr. After the protease K reaction, the samples were again heated to 95°C for 1 hr to inactivate the protease K and then washed 3 times with 0.5% TX-100 followed by 3 washes with 0.1 M sodium phosphate buffer. Samples were then resuspended in 2 ml of 0.1 M sodium phosphate buffer (pH 7.2) containing 0.04% of ß-mercaptoethanol. 1.5 mg of zymolyase (US Biologicals) was added and samples were incubated at 37**°**C for 24 hr. After zymolyase digestion, samples were washed 3 times with 0.5% TX-100 and then 3 times with H_2_O before desiccation in a speed vacuum concentrator at low temperature.

#### Solid-state NMR analysis

Spore wall solid-state NMR experiments were carried out on a 500 MHz Bruker AVANCE spectrometer using an HX magic angle spinning (MAS) probe. For analysis, 50–100 mg of dried spore wall samples were packed into 4 mm rotors. All experiments were performed at room temperature. The MAS speed was between 10 and 13 kHz to eliminate the overlap of relevant resonances with MAS sidebands. A ramped-amplitude cross polarization (CP) sequence was used to record the 1D ^13^C chemical shift spectra with proton decoupling. The experimental conditions were as follows: a 4 µs ^1^H 90° pulse was followed by 2 ms simultaneous ^13^C and ^1^H contact pulses and an acquisition time of 27 ms. The repetition delay was 3 s. 2000 to 6000 scans were recorded for each spectrum. The data were processed with exponential line broadening of 100 Hz. The carbonyl resonance of powdered glycine at 176.46 ppm relative to tetramethylsilane was used as an external ^13^C reference.

## Supporting Information

Figure S1 Solid state 13C NMR spectra for chitosan, L-tyrosine, and wild-type spore walls. Spore walls were prepared as described in Methods. Chitosan and L-tyrosine are from Sigma (Chicago, Illinois).
(TIF)

Figure S2 Complete NMR spectra for the strains shown in Figure 7. Resonances assigned to chitosan, dityrosine and component χ are indicated above the wild type spectrum. For comparison, all the spectra have been scaled to have the same height of the Chitosan C1 peak.
(TIF)

Figure S3 Two examples of wild type vegetative yeast cells co-stained with BODIPY 495/503 (green) and BODIPY TR (red).
(TIF)

Table S1 Analysis of double mutant strains. Three biological replicates were scored for each double mutant combination. Any replicate that displayed reduced dityrosine fluorescence is indicated by “−”. “+” indicates no loss of fluorescence observed. Double mutant combinations scored as interactions and used to generate Figure 2 are highlighted in yellow. n.d. = not determined.
(XLS)

Table S2 Quantitation of fluorescence images.
(DOC)

Table S3 Strains used in this study.
(DOC)

Table S4 Oligonucleotides used in this study.
(DOC)
