# Supplementary figures and images for: A Highly Redundant Gene Network Controls Assembly of the Outer Spore Wall in S. cerevisiae
Source: PLoS Genet. 2013 Aug 15;9(8):e1003700. doi: 10.1371/journal.pgen.1003700 (PMC3744438; doi:10.1371/journal.pgen.1003700)

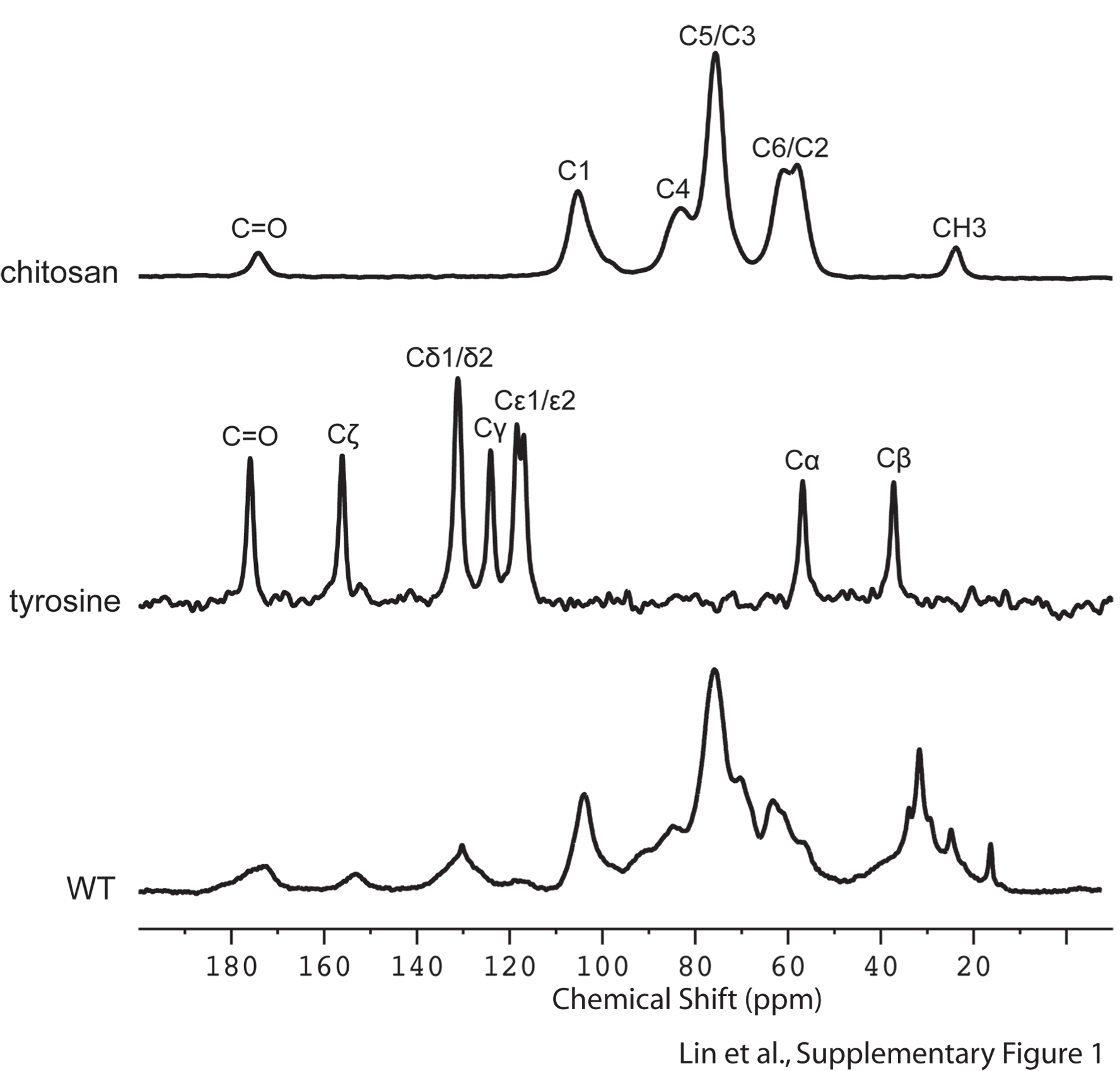

Supplement: Figure S1 — Solid state 13C NMR spectra for chitosan, L-tyrosine, and wild-type spore walls. Spore walls were prepared as described in Methods. Chitosan and L-tyrosine are from Sigma (Chicago, Illinois). (TIF) [file pgen.1003700.s001.tif]

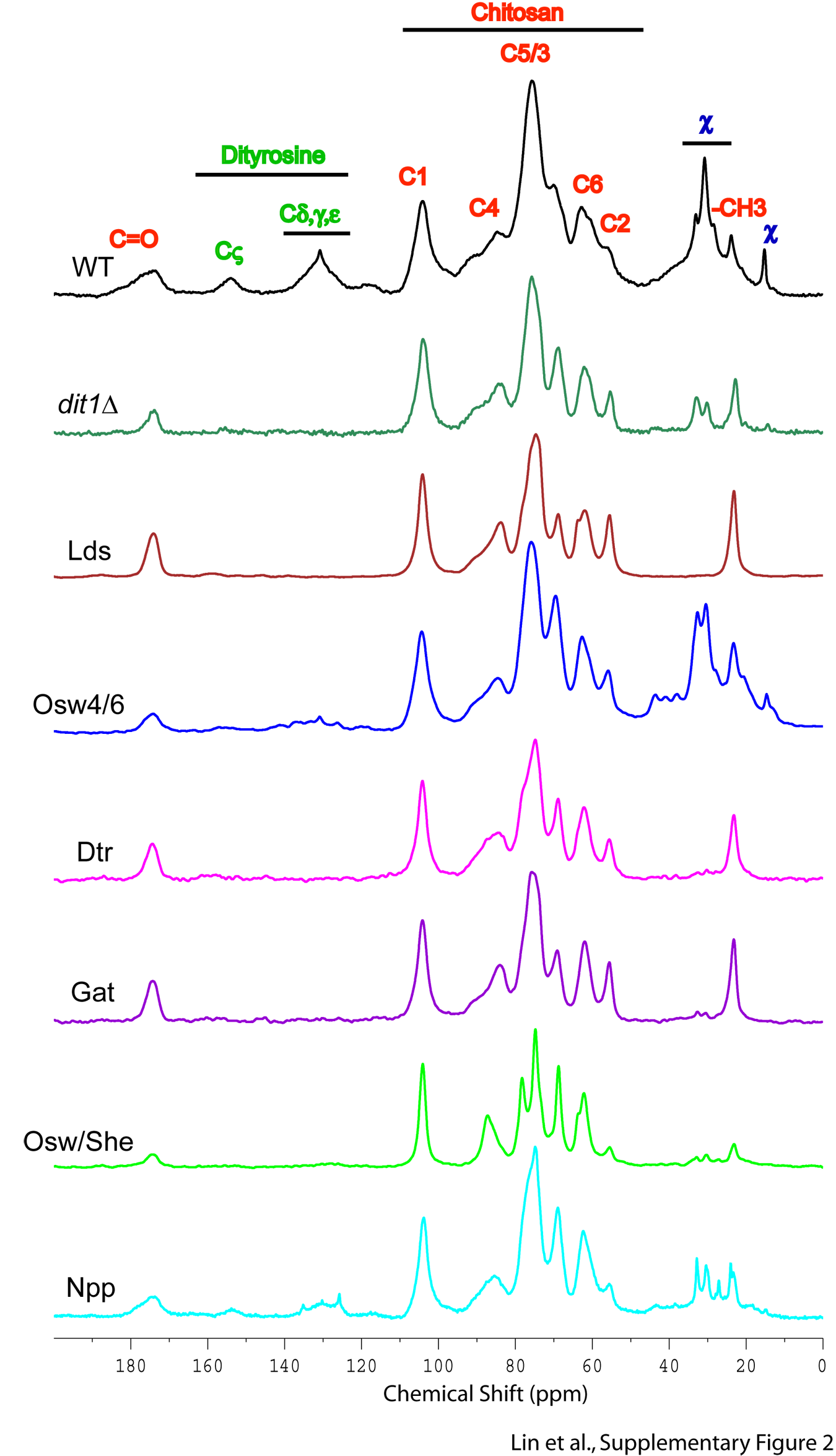

Supplement: Figure S2 — Complete NMR spectra for the strains shown in Figure 7. Resonances assigned to chitosan, dityrosine and component χ are indicated above the wild type spectrum. For comparison, all the spectra have been scaled to have the same height of the Chitosan C1 peak. (TIF) [file pgen.1003700.s002.tif]

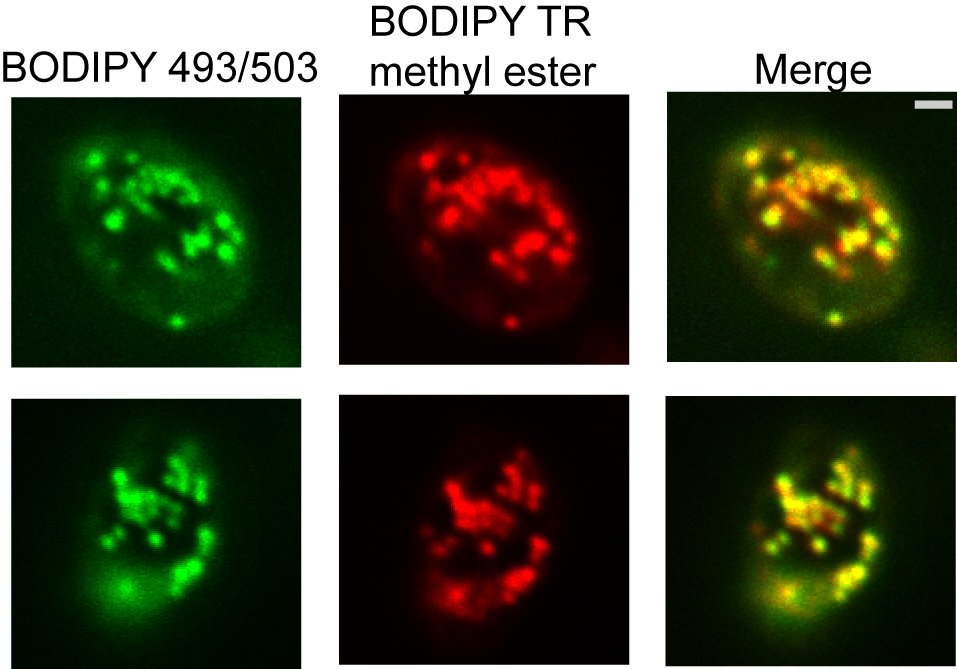

Supplement: Figure S3 — Two examples of wild type vegetative yeast cells co-stained with BODIPY 495/503 (green) and BODIPY TR (red). (TIF) [file pgen.1003700.s003.tif]
