# Supplementary material for: A Highly Redundant Gene Network Controls Assembly of the Outer Spore Wall in S. cerevisiae
Source: PLoS Genet. 2013 Aug 15;9(8):e1003700. doi: 10.1371/journal.pgen.1003700 (PMC3744438; doi:10.1371/journal.pgen.1003700)
Supplement: Table S2 — Quantitation of fluorescence images. (DOC) [file pgen.1003700.s005.doc]

Supplementary Table 2. Quantitation of fluorescence images.

% cells with pattern shown in Figure 4

Strain Eosin Y staining CFW Staining

WT 97 (n=100) 99 (100)

*cda1*Δ *cda2*Δ 100 (83) 100 (75)

*chs3*Δ 84 (94) 94 (97)

*dit1*Δ 98 (69) 82 (62)

Lds 100 (83) 76 (75)

Osw4/6 100 (78) 94 (70)

Dtr 100 (37) 86 (36)

Gat 100 (33) 97 (34)

Osw/She 100 (44) 74 (42)

Npp 100 (100) 60 (98)

% cells with pattern shown in Figure 8

Marker Meiosis II Post-Meiotic

Lds1 GFP 100 (n=90) 100 (120)

Lds2-GFP 100 (87) 100 (72)

Lds3-GFP 100 (101) 100 (60)

BODIPY TR 100 (86) 100 (80)
