# Supplementary material for: A Highly Redundant Gene Network Controls Assembly of the Outer Spore Wall in S. cerevisiae
Source: PLoS Genet. 2013 Aug 15;9(8):e1003700. doi: 10.1371/journal.pgen.1003700 (PMC3744438; doi:10.1371/journal.pgen.1003700)
Supplement: Table S3 — Strains used in this study. (DOC) [file pgen.1003700.s006.doc]

**Supplementary Table 3**

**Yeast strains used in this study**

Strain Genotype Source

AN117-4B *MATα ura3 leu2 his3∆SK trp1*Δ*::hisG arg4-NspI lys2 hoΔ::LYS2 rme1*Δ*::LEU2*  Neiman et al., 2000

AN117-16D *MAT****a*** *ura3 leu2 his3∆SK trp1Δ::hisG lys2 ho*Δ*::LYS2* Neiman et al., 2000

AN120 *MAT****a****/MATα ura3/ura3 leu2/leu2 his3∆SK/his3∆SK trp1Δ::hisG/trp1Δ::hisG ARG4/arg4-NspI lys2/lys2*  Neiman et al., 2000

*ho*Δ*::LYS2/ho*Δ*::LYS2 RME1/rme1*Δ*::LEU2*

AN262 *MAT****a****/MATα ura3/ura3 leu2/leu2 his3∆SK/his3∆SK trp1*Δ*::hisG/trp1*Δ*::hisG ARG4/arg4-NspI lys2/lys2*  Coluccio et al., 2004

*ho*Δ*::LYS2/ho*Δ*::LYS2 RME1/rme1*Δ*::LEU2 chs3*Δ*::HIS3MX6/chs3*Δ*::HIS3MX6*

AN264 *MAT****a****/MATα ura3/ura3 leu2/leu2 his3∆SK/his3∆SK trp1*Δ*::hisG/trp1*Δ*::hisG ARG4/arg4-NspI lys2/lys2*  Coluccio et al., 2004

*ho*Δ*::LYS2/ho*Δ*::LYS2 RME1/rme1*Δ*::LEU2 dit1*Δ*::HIS3MX6/dit1*Δ*::HIS3MX6*

K8409 *MATa/MATa HO/HO his3/his3 trp1/trp1 lys2/lys2 LEU2::P_URA3_-tetR-GFP/LEU2::P_URA3_-tetR-* Rabitsch et al., 2001

*GFP REC8::HA3-URA3/REC8::HA3-URA3 URA3::tetO224/URA3::tetO224*

BY4741 *MAT****a*** *his3*Δ*1 leu2*Δ*0 met15*Δ*0 ura3*Δ*0* (Winzeler et al. 1999)

MYA-1801 as K8409, plus *lds1*Δ*::HIS3MX6/lds1*Δ*::HISMX6* Rabitsch et al., 2001

MYA-1810 as K8409, plus *dtr1*Δ*::HIS3MX6/dtr1*Δ*::HISMX6* Rabitsch et al., 2001

MYA-1867 as K8409, plus *npp2*Δ*::HIS3MX6/npp2*Δ*::HISMX6* Rabitsch et al., 2001

MYA-1890 as K8409, plus *osw7*Δ*::HIS3MX6/osw7*Δ*::HISMX6* Rabitsch et al., 2001

MYA-1941 as K8409, plus *gat4*Δ*::HIS3MX6/gat4*Δ*::HISMX6* Rabitsch et al., 2001

MYA-1976 as K8409, plus *gat3*Δ*::HIS3MX6/gat3*Δ*::HISMX6* Rabitsch et al., 2001

MYA-2046 as K8409, plus *lds2*Δ*::HIS3MX6/lds2Δ::HISMX6* Rabitsch et al., 2001

LDS1-GFP as BY4741, plus *LDS1::GFP::HIS3* Huh et al., 2003

LDS2-GFP as BY4741, plus *LDS2::GFP::HIS3* Huh et al., 2003

RRT8-GFP as BY4741, plus *RRT8::GFP::HIS3* Huh et al., 2003

CL6 *MAT****a****/MATα HO/HO leu2/leu2 lys2/lys2 URA3::tet0224/URA:3:tet0224 lds1*Δ*::HIS3/*  This study

*lds1*Δ*::HIS3 rrt8*Δ*::kanMX6/rrt8*Δ*::kanMX6 lds2*Δ*::HphMX6/*

*lds2*Δ*::HphMX6*

CL7 *MAT****a****/ MATα HO/HO leu2/leu2 lys2/lys2 URA3::tet0224/ URA3::tet0224 dtr1*Δ*::HIS3/dtr1*Δ*::HIS3*  This study

*qdr3*Δ*::kanMX6/qdr*3Δ*::kanMX6 qdr1*Δ*::HphMX6/qdr1*Δ*::HphMX6*

CL11 *MAT****a****/ MATα ho/ho ura3/ura3 leu2/leu2 trp1-hisG/trp1-hisG his3*Δ*SK/his3*Δ*SK lys2/lys2*  This study

*RME1/rme1*Δ*::LEU2 ARG4/arg4-Nsp1 ho*Δ*::LYS2/ho*Δ*::LYS2 osw4,6*Δ*::HIS3/osw4,6*Δ*::HIS3*

*URA3::P_SPR1_-GFP-OSW4(URA3)/ URA3::P_SPR1_-GFP-OSW4(URA3)*

CL15 *MAT****a****/MATα HO/HO leu2/leu2 trp1/trp1 lys2/lys2 URA3::tet0224/URA3::tet0224 gat4*Δ*::HIS3/*  This study

*gat4*Δ*::HIS3 gat3*Δ*::kanMX6/ gat3*Δ*::kanMX6*

CL26 *MAT****a****/MATα HO/HO leu2/leu2 URA3::tet0224/ URA3::tet0224*  This study

*osw7*Δ*::HIS3/osw7*Δ*::HIS3 she10*Δ*::kanMX6/she10*Δ*::kanMX6*

CL35 *MAT****a****/MATα ura3/ura3 leu2/leu2 his3∆SK/his3∆SK trp1Δ::hisG/trp1*Δ*::hisG ARG4/arg4-NspI lys2/lys2*  This study

*hoΔ::LYS2/ho*Δ*::LYS2 RME1/rme1Δ::LEU2 osw4,6*Δ*::HIS3MX6/osw4,6*Δ*::HIS3MX6*

CL38 *MAT****a****/MATα HO/HO his3/his3 trp1/trp1 LEU2::P_URA3_-tetR-GFP/LEU2::P_URA3_-tetR-GFP*  This study

*URA3::tetO224/URA3::tetO224 rrt8*Δ*::kanMX6/rrt8*Δ*::kanMX6*

CL43 *MAT****a****/MATα HO/HO his3/his3 lys2/lys2 LEU2::P_URA3_-tetR-GFP/LEU2::P_URA3_-tetR-GFP*  This study

*URA3::tetO224/URA3::tetO224 qdr1*Δ*::kanMX6/qdr1*Δ*::kanMX6*

CL44 *MAT****a****/MATα HO/HO his3/his3 trp1/trp1 leu2/leu2 URA3::tetO224/URA3::tetO224 qdr3*Δ*::kanMX6/*  This study

*qdr3*Δ*::kanMX6*

CL47 *MATa/MATα HO/HO his3/his3 trp1/trp1 LEU2::P_URA3_-tetR-GFP/LEU2::P_URA3_-tetR-GFP*  This study

*URA3::tetO224/URA3::tetO224 she10*Δ*::kanMX6/she10*Δ*::kanMX6*

CL50 *MATa/MATα ura3/ura3 leu2/leu2 his3∆SK/his3∆SK trp1Δ::hisG/trp1*Δ*::hisG ARG4/arg4-NspI lys2/lys2*  This study

*hoΔ::LYS2/ho*Δ*::LYS2 RME1/rme1*Δ*::LEU2 cda1,2*Δ*::HygB/cda1,2*Δ*::HygB*

CL52 *MAT****a****/MATα ura3/ura3 leu2/leu2 his3∆SK/his3∆SK trp1*Δ*::hisG/trp1*Δ*::hisG ARG4/arg4-NspI lys2/lys2*  This study

*ho*Δ*::LYS2/ho*Δ*::LYS2 RME1/rme1*Δ*::LEU2 osw4*Δ*::HIS3MX6/osw4*Δ*::HIS3MX6*

CL54 *MAT****a****/MATα ura3/ura3 leu2/leu2 his3∆SK/his3∆SK trp1Δ::hisG/trp1*Δ*::hisG ARG4/arg4-NspI lys2/lys2*  This study

*ho*Δ*::LYS2/ho*Δ*::LYS2 RME1/rme1*Δ*::LEU2 osw6*Δ*::HIS3MX6/osw6*Δ*::HIS3MX6*

CL57 *MAT****a****/MATα HO/HO leu2/leu2 TRP1/TRP1 lys2/lys2 URA3:tet0224/ URA3:tet0224*  This study

*npp2*Δ*::HIS3 npp1*Δ*::kanMX6/npp2*Δ*::HIS3 npp1*Δ*::kanMX6*

CL59 *MAT****a****/MATα HO/HO leu2/leu2 URA3::tetO224/URA3::tetO224 npp1*Δ*::kanMX6/npp1*Δ*::kanMX6*  This study

CL62 *MAT****a*** *his3*Δ*1 leu2*Δ*0 met15*Δ*0 ura3*Δ*0 cyh2* This study
