# Supplementary material for: A Highly Redundant Gene Network Controls Assembly of the Outer Spore Wall in S. cerevisiae
Source: PLoS Genet. 2013 Aug 15;9(8):e1003700. doi: 10.1371/journal.pgen.1003700 (PMC3744438; doi:10.1371/journal.pgen.1003700)
Supplement: Table S4 — Oligonucleotides used in this study. (DOC) [file pgen.1003700.s007.doc]

| Primer | Sequence |
| --- | --- |
| LDS2-KO-F | TCTTAGTTATAGCATTAGAACTATAAGAGCTGCTAAGACACCAAGCAAACCGGATCCCCGGGTTAATTAA |
| LDS2-KO-R | TGTAAACATATAATGAATATATAAATTGGTATGTAAAAGAGTTGCGAGCTGAATTCGAGCTCGTTTAAAC |
| QDR1-KO-F | TTAATAAAAAAATAACAGATAGCTCATGAAGAGACTTCTATAAGTAAATCCGGATCCCCGGGTTAATTAA |
| QDR1-KO-R | GTTTCTGGAAAGTGGGGGCAGAGACTTTTTAGTTTTACGACTTTTTTTCTGAATTCGAGCTCGTTTAAAC |
| ANO262-A | GCAGCTATGCCGGTGCATGCGCACAGTATAATGGACTTAAACTGCCTTAGTCGGATCCCCGGGTTAATTAA |
| ANO262-B | ACAAAACAATAAGTGAAGATAAAAGAGCCTAGTGAAGTAAACTAATTCAAGAATTCGAGCTCGTTTAAAC |
| ANO263-A | TGAGATTAAACAATGTACAGGAGTGGCAAAAACAAAATCGGCAACATTTTCGAATTCGAGCTCGTTTAAAC |
| ANO263-B | TGCTTTTCCAATAAAAATCATAATCACAGCCATAGTCACAAAACTATAAGGGATCCCCGGGTTAATTAA |
| CDA1&2-KO-F | GTAAGCAAAGTGGAATATAGAGAAACAAAATTAAGTGATAGATTTACTTTTACACAAGAAAAGAGCGGATCCCCGGGTTAATTAA |
| CDA1&2-KO-R | CTAAAGCAATTTCTTTTTAAATTGTAATTTAATTCTTCCTTATTTTCTTCAATTCCCTGAAAAGAATTCGAGCTCGTTTAAAC |

Table S4. Primers used in this study.
